# Transformations of summary statistics as input in meta-analysis for linear dose-response models on a logarithmic scale: a methodology developed within EURRECA

**DOI:** 10.1186/1471-2288-12-57

**Published:** 2012-04-25

**Authors:** Olga W Souverein, Carla Dullemeijer, Pieter van `t Veer, Hilko van der Voet

**Affiliations:** 1 Division of Human Nutrition, Wageningen University and Research Centre, P.O. Box 8129, 6700, EV Wageningen, the Netherlands; 2 Biometris, Wageningen University and Research Centre, P.O. Box 100, 6700, AC Wageningen, the Netherlands

**Keywords:** Methodology, Dose–response, Meta-analysis, EURRECA

## Abstract

**Background:**

To derive micronutrient recommendations in a scientifically sound way, it is important to obtain and analyse all published information on the association between micronutrient intake and biochemical proxies for micronutrient status using a systematic approach. Therefore, it is important to incorporate information from randomized controlled trials as well as observational studies as both of these provide information on the association. However, original research papers present their data in various ways.

**Methods:**

This paper presents a methodology to obtain an estimate of the dose–response curve, assuming a bivariate normal linear model on the logarithmic scale, incorporating a range of transformations of the original reported data.

**Results:**

The simulation study, conducted to validate the methodology, shows that there is no bias in the transformations. Furthermore, it is shown that when the original studies report the mean and standard deviation or the geometric mean and confidence interval the results are less variable compared to when the median with IQR or range is reported in the original study.

**Conclusions:**

The presented methodology with transformations for various reported data provides a valid way to estimate the dose–response curve for micronutrient intake and status using both randomized controlled trials and observational studies.

## Background

Meta-analysis of the association between micronutrient intake and biochemical proxies for micronutrient status or function is needed when setting micronutrient recommendations. Information on this association may come from randomized controlled trials as well as from observational studies. In a randomized trial subjects are randomized to receive either the intervention treatment or the control treatment, and a meta-analysis of such studies will usually provide a mean difference in micronutrient status between placebo and intervention groups, answering the question whether the biochemical status marker responds to the dietary intake of a micronutrient [1-3]. However, this analysis does not provide an estimate of the slope of the dose–response relationship. On the other hand, a meta-analysis of observational studies provides an estimate of the slope of the dose–response relation, but observational studies are hampered by for instance measurement error in the intake estimates, which causes bias in the reported association [4-6].

Ideally, information from observational studies and randomized controlled trials should be compared or even combined in a single meta-analysis to ensure that all reported information is taken into account over a broad range of intake. This requires that the summary statistics reported in individual studies are transformed into estimates of the dose–response relation. Since both intake and status are continuous variables, this estimate is actually an estimate of the regression coefficient of the linear regression of micronutrient status on micronutrient intake. The individual estimates of the dose–response regression coefficient may then be combined in a meta-analysis.

The statistical combination of study results may be complicated by the variety of ways that individual studies report the summary statistics. The results from randomized controlled trials as well as the baseline summary statistics of micronutrient intake and status may be reported as means, medians or geometric means. Variability is often reported as standard deviations, standard errors, interquartile ranges (IQR), ranges or confidence intervals (CI). In observational studies the relation between intake and status can be reported as a Pearson correlation coefficient, a Spearman rank correlation coefficient or a regression coefficient. In addition, either the intake variable or the status variable or both could have been logarithmically transformed before the correlation or association was calculated. All these different ways of reporting need to be standardized before meta-analysis is even possible.

This paper gives an overview of transformation methods to algebraically derive an estimate from each study of the regression coefficient (slope, b) and its standard error (se(b)), for studies that do not directly report these. The methods are validated by comparing the calculated values with theoretical values in a small-scale simulation study.

## Methods

In order to derive transformations we assume a bivariate normal distribution on the log-scale for intake and status of an individual person. The log-scale was chosen because both intake and status values are always above zero, and the observed distributions of the micronutrient variables are often right-skewed. Moreover, as the true shape of the dose–response curve is usually unknown the linear relation between logarithmically transformed quantities provides the simplest approximation.More in detail, for the dose–response meta-analysis of observational studies we assume that $\xi_{0}$ (intake of micronutrient) and $\eta_{0}$ (status or continuous health outcome) are log-normally distributed. The assumption of bivariate normality entails a linear association between $\xi =\text{ln}(\xi_{0})$and $\eta =\text{ln}(\eta_{0})$, where ln denotes the natural logarithm. Note that we use the Greek letters *ξ* and *η* for the theoretical values of intake and status/response, and the Latin letters X and Y for the observed values of these variables. Furthermore, we reserve letters without subscript (e.g. X and Y) for values expressed on the ln-scale, and use letters with subscript 0 (e.g., X_0_ and Y_0_) for values expressed on the absolute (i.e., original) scale.

**Figure 1:**
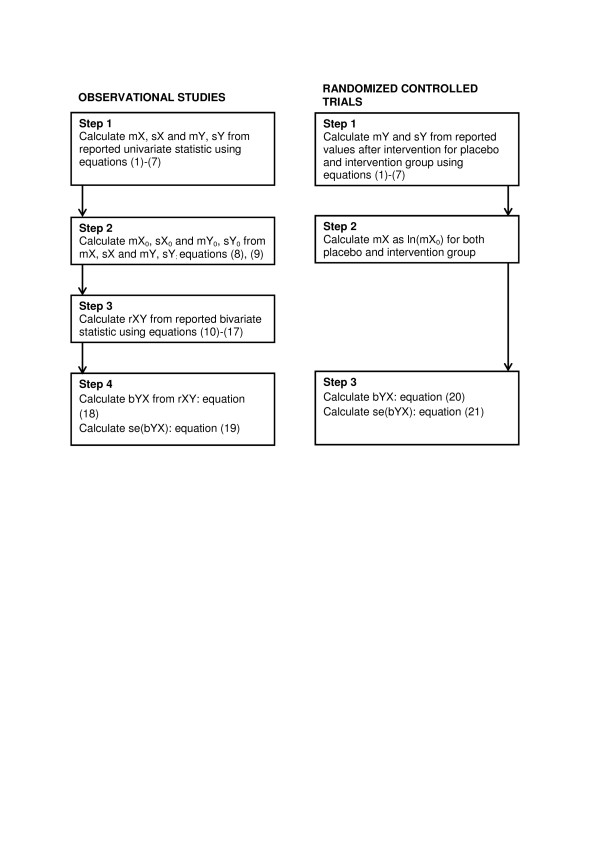
**Flowchart indicating the process of data transformations.** m, mean, s, standard deviation, b, regression coefficient, r, correlation coefficient. X indicates intake of micronutrient and Y indicates a proxy for micronutrient status or continuous health outcome. Capital letters without subscript are used for values expressed on the ln-scale and small letters with subscript 0 for values expressed on the absolute scale.

The process of data transformations to obtain the required statistics from what is reported in observational studies, consists of four steps (Figure 1). The first step is to obtain the mean of X (mX) and Y (mY) and the standard deviation of X (sX) and Y (sY). Secondly, the mean of X_0_ (mX_0_) and Y_0_ (mY_0_) and the standard deviation of X_0_ (sX_0_) and Y_0_ (sY_0_) are calculated when needed for the calculations in step 3. In this third step the correlation coefficient of the association between X and Y (rXY) is calculated from the reported data. In the last step, the regression coefficient of the linear regression from Y on X (bYX) is calculated from rXY, and the se(bYX) is calculated from rXY, sY, sX and the sample size (n). For reports on randomized controlled trials, the process consists of three steps. In the first step, mY and sY are obtained for both intervention and placebo group. In the second step, mX is obtained, and in the last step, bYX and se(bYX) are calculated. The equations for all these transformations are given below.

### Univariate transformations

First, we describe how the univariate statistics of the normal distributions at the ln-scale can be obtained from various reported statistics. We present formulas for mX and sX, which of course can also be used similarly for mY and sY in observational studies. For randomized controlled trials the situation is different, because the variation in X is artificial and is not described by a normal distribution. Therefore, the transformations should be used only to obtain mY and sY in the intervention and placebo groups separately. In most trials the within-group variation in X will be ignorable compared with the difference between the groups, consequently mX is calculated simply as mX_con_ = ln(mX_0_con_) for the placebo group and as mX_int_ = ln(mX_0_int_) the intervention group.

For these transformations, we assume that  ξ is normally distributed with parameters $\mu_{\xi }$and $\sigma_{\xi }$. For a lognormal distribution the mean on the absolute scale, $\mu_{\xi_{0}}$, is given by $\mu_{\xi_{0}}=\exp\left(\mu_{\xi }+0.5\;\sigma_{\xi }^{2}\right)$ and the standard deviation on the absolute scale, $\sigma_{\xi_{0}}$, is given by $\sigma_{\xi_{0}}=\exp\left(\mu_{\xi }+0.5\sigma_{\xi }^{2}\right)\sqrt{\exp\left(\sigma_{\xi }^{2}\right)-1}$. It follows that when the mean (mX_0_) and the standard deviation (sX_0_) are reported, mX can be calculated as:

(12) $$\text{mX}={\text{ln(mX}}_{0})-0{\text{.5sX}}^{2}$$

where

(2) $$\text{sX}=\sqrt{\text{ln}\left(1+{\left(\frac{{\text{sX}}_{0}}{{\text{mX}}_{0}}\right)}^{2}\right)}$$

The exponential function of the mean of the lognormal distribution is equal to the median on the absolute scale. Therefore, when the median (medX_0_) has been reported on the absolute scale, mX is calculated as:

(14) $$\text{mX}={\text{ln(medX}}_{0})$$

As a measure of variability an IQRx or range (rangex) is often reported together with the median or mean. The IQR is the difference between the third quartile Q_3_ and first quartile Q_1_ (the 75^th^ percentile and the 25^th^ percentile). Basically, there are two cases. If the lower and upper limits are reported as such, the difference between the ln-transformed limits may be equated to an appropriate multiple of the standard deviation sX. On the other hand, if only the IQR or range is reported as such, the derivation is more complex. When IQRX_0_is reported together with the median, the relation between these and sX is given by ${\text{IQRX}}_{0}={\text{medX}}_{0}\times \left[\exp\left(\text{z}\cdot \text{sX}\right)-\exp\left(-\text{z}\cdot \text{sX}\right)\right]$, where z represents the appropriate percentage point in the standard normal distribution (i.e., z_0.75_ = 0.6745).

In this case sX may be calculated as

(16) $$\text{sX}=\frac{\ln\left[\frac{1}{2}\times \left(\frac{{\text{IQRX}}_{0}}{{\text{medX}}_{0}}+\sqrt{{\left(\frac{{\text{IQRX}}_{0}}{{\text{medX}}_{0}}\right)}^{2}+4}\right)\right]}{\text{z}}$$

When the IQR is reported together with the mean no explicit formula exists to derive sX. Therefore, to obtain an estimate of sX from these quantities a nonlinear function optimization is employed to find the value of sX for which the following equation holds

(17) $${\text{IQRX}}_{0}={\text{mX}}_{0}\times \exp\left(-0.5{\text{sX}}^{2}\right)\times \left[\exp\left(\text{z}\cdot \text{sX}\right)-\exp\left(-\text{z}\cdot \text{sX}\right)\right]\text{.}$$

When the lower and upper bounds of the IQR (i.e., Q_1_(X_0_) and Q_3_(X_0_) respectively) are reported, rather than the difference, sX may be calculated as $\text{sX}=\left[Q_{3}(X)-Q_{1}(X)\right]/2\text{z.}$

The range is the difference between the maximum and the minimum value of the data. Equations (4) and (5) may be similarly used when the range is reported, but here we consider that the minimum and the maximum represent the lower and upper (1/n) fraction of the dataset of n observations. Therefore we expect a fraction p = 1-1/(2n) below the minimum and the same fraction above the maximum, and in the equations above we need to use z_p_. For example, in a dataset with n = 100 we use z_0.995_ = 2.576.

The geometric mean (gm) of the lognormal distribution is equal to exp(mX), and is most often reported in papers together with the 95% confidence limits. mX and sX are obtained for these quantities using:

(19) $$\text{mX}={\text{ln(gmX}}_{0}),$$

(20) $$\text{sX}=\sqrt{\text{n}}\times \frac{\left[\text{ln}\left({\text{X}}_{0\text{,upp}}\right)-\text{ln}\left({\text{X}}_{0\text{,low}}\right)\right]}{2\cdot {\text{z}}_{0\cdot 975}}$$

where X_0,upp_ is the upper limit, X_0,low_ is the lower limit of the 95% confidence interval and z_0.975_ = 1.96 represents the 97.5th percentage point in the standard normal distribution.

Then in step 2 for observational studies, mx and sx are calculated in case these estimates were not already available. These statistics at the original scale may be needed in the bivariate transformations described below. The equations are:

(21) $${\text{mX}}_{0}=\text{exp(mX}+0{\text{.5sX}}^{2})$$

(22) $${\text{sX}}_{0}={\text{mX}}_{0}\times \sqrt{\text{exp}\left({\text{sX}}^{2}\right)-1}$$

### Bivariate transformations (to obtain regression or correlation coefficients)

For observational studies, the next step is to obtain an estimate of the correlation between X and Y (rXY). The equations below can be used to obtain rXY from reported correlation and regression coefficients taking into account the possibility that either X_0_, log_10_(X_0_), X, Y_0_, log_10_(Y_0_) or Y was used for the originally reported statistic.

When a study reports the association as a Spearman rank correlation coefficient (r_S_), rXY is calculated as

(23) $$\text{rXY}={\text{r}}_{\text{s}}$$

Another option is that the association between X_0_ and Y_0_ is reported as a regression coefficient (bY_0_X_0_). In that case the correlation coefficient, rX_0_Y_0_, is calculated first using

(24) $${\text{rX}}_{0}{\text{Y}}_{0}={\text{bY}}_{0}{\text{X}}_{0}\times \frac{{\text{sX}}_{0}}{{\text{sY}}_{0}}$$

and then rXY is calculated using the following equation which was derived from Johnson & Kotz [7]:

(25) $$\text{rXY}=\frac{\text{ln}\left\{\;1+{\text{rX}}_{0}{\text{Y}}_{0}\times \sqrt{\left[\text{exp}\left({\text{sX}}^{2}\right)-1\right]\times \left[\text{exp}\left({\text{sY}}^{2}\right)-1\right]}\right\}}{\text{sX}\times \text{sY}}$$

This formula (12) is also used when the Pearson product–moment correlation coefficient rX_0_Y_0_ is directly reported in a paper.

For observational studies that report the regression coefficient between Y_0_ and X, the correlation coefficient, rXY_0_, is calculated using

(26) $${\text{rXY}}_{0}={\text{bY}}_{0}\text{X}\times \frac{\text{sX}}{{\text{sY}}_{0}}$$

When log_10_(X_0_) is used instead of X, sX is replaced by sX/ln(10) in formula (13).

Then rXY is calculated using the following equation [8,9]:

(27) $$\text{rXY}={\text{rXY}}_{0}\times \frac{\sqrt{\left[\text{exp}\left({\text{sY}}^{2}\right)-1\right]}}{\text{sY}}$$

This formula (14) is also used when rXY_0_ is reported directly or when the Pearson product–moment correlation coefficient is reported between log_10_(X_0_) and Y_0_.

When the regression coefficient between Y and X_0_ is reported in an observational study, the regression coefficient, rX_0_Y, is calculated using

(28) $${\text{rX}}_{0}\text{Y}={\text{bYX}}_{0}\times \frac{{\text{sX}}_{0}}{\text{sY}}$$

When log_10_(Y_0_) is used instead of Y, sY is replaced by sY/ln(10) in formula (15).

Using rX_0_Y or the directly reported Pearson product–moment correlation coefficient between X_0_ and log_10_(Y_0_) or Y in an observational study, rXY is calculated using [8,9]:

(29) $$\text{rXY}={\text{rX}}_{0}\text{Y}\times \frac{\sqrt{\left[\text{exp}\left({\text{sX}}^{2}\right)-1\right]}}{\text{sX}}$$

When the regression coefficient between X and Y is reported, rXY is calculated as

(30) $$\text{rXY}=\text{bYX}\times \frac{\text{sX}}{\text{sY}}$$

### Calculation of dose–response regression coefficient

In the last step, for both observational studies and randomized controlled trials, we need to obtain bYX and se(bYX). For observational studies, the required regression coefficient bYX is calculated from the correlation coefficient:

(31) $$\text{bYX}=\text{rXY}\times \frac{\text{sY}}{\text{sX}}$$

and the corresponding standard error (se(bYX)) is calculated as

(32) $$\text{se(bYX})=\sqrt{\frac{sY^{2}\times \left(1-rXY^{2}\right)}{\left(N-2\right)\times sX^{2}}}$$

For randomized controlled trials, the required regression coefficient bYX is calculated as:

(33) $$\text{bYX}=\frac{{\text{mY}}_{\text{int}}-{\text{mY}}_{\text{con}}}{{\text{mX}}_{\text{int}}-{\text{mX}}_{\text{con}}}$$

where ‘int’ indicates the intervention group and ‘con’ indicates the control or placebo group. The corresponding standard error is calculated as:

(34) $$\text{se}\left(\text{bYX}\right)=\sqrt{\left(\frac{\left({\text{N}}_{\text{con}}-1\right)\times {\text{sY}}_{\text{con}}{}^{2}+\left({\text{N}}_{\text{int}}-1\right)\times {\text{sY}}_{\text{int}}{}^{2}}{\left({\text{N}}_{\text{con}}+{\text{N}}_{\text{int}}-2\right)}\right)\times \left(\frac{1}{{\text{N}}_{\text{con}}}+\frac{1}{{\text{N}}_{\text{int}}}\right)\times \left(\frac{1}{{\left({\text{mX}}_{\text{int}}-{\text{mX}}_{\text{con}}\right)}^{2}}\right)}$$

### Simulation study

A simulation study was conducted to validate the performance of the transformations given in this paper. Bivariate lognormal data (X,Y) were simulated where X ~ Normal(1.60,0.85^2^) and Y ~ Normal(5.70,0.45^2^). Parameter values were based on values of vitamin B12 intake (X) and serum/plasma vitamin B12 (Y) [10-13]. Different strengths of the correlation between X and Y were simulated, namely 0.1, 0.5 and 0.9.

A sample of individuals (with sample size 100, 200 or 500) was randomly drawn, and values that represent different often used reporting options were calculated from this sample, namely the mean and SD, the median and IQR, the median and range and the geometric mean and 95% CI (all summary statistics on the absolute scale). Also, the correlation and regression coefficients of X and Y expressed in different scales were calculated. These ‘reported’ values were rounded to two decimal places. From these ‘reported’ values, the parameter estimates mX, mY, sX, sY and rXY were calculated using the transformations described in this paper. This process was repeated 1000 times.

## Results

Table 1 shows the simulation results for the univariate statistics. On average the calculated values of mX and mY are almost the same as the true values, indicating that no important bias is present in these calculations. As expected, the 95% CI of the simulations is smaller for the simulations with a sample size of 500 than for the simulations with a sample size of 200 or 100. For sX and sY, the estimates are most precise when a geometric mean with a 95% CI is reported, and least precise when a median with a range is reported.

**Table 1:** Simulation results for mX, sX, mY and sY

	**n**	**mX**	**sX**	**mY**	**sY**
True		1.6	0.85	5.7	0.45
Mean, SD	100	1.6 (1.4-1.8)	0.82 (0.65-1.06)	5.7 (5.6-5.8)	0.45 (0.37-0.53)
	200	1.6 (1.4-1.7)	0.83 (0.70-1.03)	5.7 (5.6-5.8)	0.45 (0.40-0.51)
	500	1.6 (1.5-1.7)	0.84 (0.75-0.98)	5.7 (5.7-5.7)	0.45 (0.42-0.49)
Median, IQR	100	1.6 (1.4-1.8)	0.84 (0.63-1.10)	5.7 (5.6-5.8)	0.44 (0.35-0.56)
	200	1.6 (1.5-1.8)	0.85 (0.70-1.02)	5.7 (5.6-5.8)	0.45 (0.38-0.53)
	500	1.6 (1.5-1.7)	0.85 (0.76-0.95)	5.7 (5.7-5.7)	0.45 (0.40-0.50)
Median, range	100	1.6 (1.4-1.8)	0.83 (0.58-1.14)	5.7 (5.6-5.8)	0.44 (0.32-0.60)
	200	1.6 (1.5-1.8)	0.83 (0.63-1.12)	5.7 (5.6-5.8)	0.44 (0.35-0.58)
	500	1.6 (1.5-1.7)	0.83 (0.68-1.06)	5.7 (5.7-5.7)	0.44 (0.36-0.56)
Gm, 95% CI	100	1.6 (1.4-1.8)	0.85 (0.73-0.97)	5.7 (5.6-5.8)	0.45 (0.38-0.51)
	200	1.6 (1.5-1.7)	0.85 (0.77-0.94)	5.7 (5.6-5.8)	0.45 (0.41-0.49)
	500	1.6 (1.5-1.7)	0.85 (0.80-0.90)	5.7 (5.7-5.7)	0.45 (0.42-0.48)

**Figure 2:**
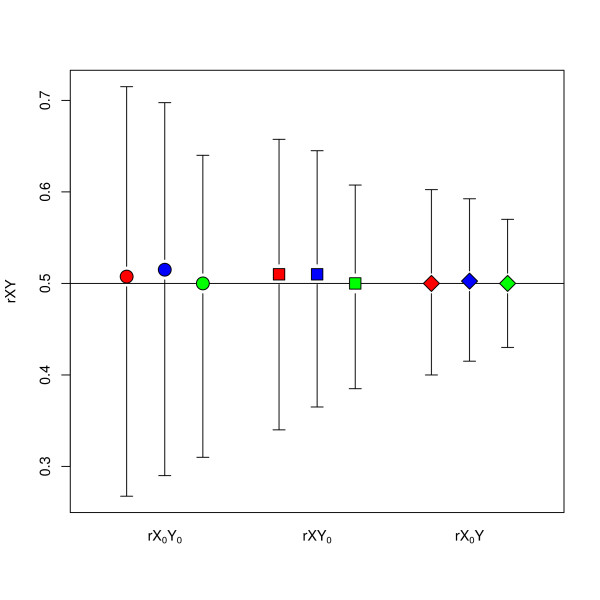
**Simulation results for rXY where the true rXY was 0.5.** Circles indicate that the reported bivariate statistic was rX_0_Y_0_, squares indicate rXY_0_ and diamonds indicate rX_0_Y. Bars represent 95% confidence intervals. In each group the three bars from left to right are for sample sizes of 100, 200 and 500 individuals, respectively.

**Figure 3:**
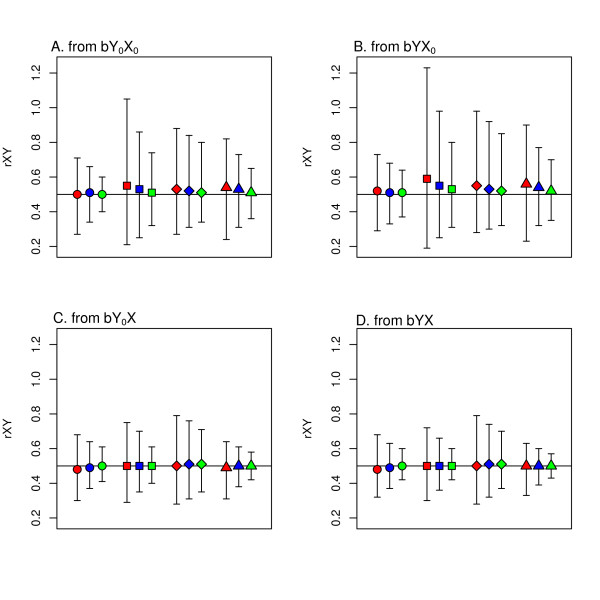
**Simulation results for rXY from different reporting options where the true rXY was 0.5. A) b from linear regression of Y**_**0**_**on X**_**0**_**; B) b from linear regression of Y on X**_**0**_**; C) b from linear regression of Y**_**0**_**on X; D) b from linear regression of Y on X and from the linear regression of log**_**10**_**(Y**_**0**_**) on log**_**10**_**(X**_**0**_**).** Circles indicate that the reported univariate statistic was mean + SD, squares indicate median and IQR, diamonds indicate median and range, and triangles indicate geometric mean. Bars represent 95% confidence intervals. In each group the three bars from left to right are for sample sizes of 100, 200 and 500 individuals, respectively.

Figure 2 shows the simulation results when a correlation coefficient is reported, and Figure 3 shows the simulation results when a linear regression coefficient is reported. Both these figures show the simulation results with true rXY = 0.5. Results are similar for true rXY = 0.9 and true rXY = 0.1 (data not shown). For the situation in which a correlation coefficient is the reported bivariate statistic, there is no difference for the four univariate reporting options. Therefore, these results are pooled in Figure 2.

None of the combinations of univariate and bivariate reporting options shows evidence of bias with the average of the simulations almost equal to the true value. The width of the confidence interval indicates the variability of the simulations. Because there is no appreciable bias, a smaller CI width indicates that the individual simulations are closer to the true correlation. The accuracy is best when rX_0_Y is reported and worst when rX_0_Y_0_ is reported. As expected, the accuracy is also better when the sample size is larger. Figure 3 shows that the CI is wider when the reported univariate statistics are the median and IQR or median and range. The larger variation in the results for the transformation from bYX_0_ (Figure 3B) compared with the variation in the results from bY_0_X (Figure 3C) is caused by the fact the X was simulated with larger standard deviation than Y.

### Example

To illustrate the methodology some examples of its use on real data for vitamin B12 are reported in Table 2 (observational studies [14,15]) and Table 3 (randomized controlled trials [16,17]). The tables show the statistics as reported in the studies and the statistics that are calculated using the different equations presented in this paper (which are entitled ‘required statistics’ in the tables).

**Table 2:** Example statistics for observational studies on vitamin B12 intake (X) and vitamin B12 status (Y)

**Reference**	**Observed univariate statistics**				**Observed bivariate statistic**				**Required statistics**				
	**Type X_0_ and Y_0_**	**X_0_**	**Y_0_**	**n**	**Association**		**mX**	**sX**	**mY**	**sY**	**rXY**	**bYX**	**se(bYX)**
[14]	Mean, SD	9.3, 9.3	330, 140	177	rX_0_Y_0_	0.16	1.88	0.83	5.72	0.41	0.19	0.09	0.04
[15]	gm, 95% CI	7.3, 7.1-7.5	354, 348-360	1329	r_s_	0.19	1.99	0.51	5.87	0.32	0.19	0.12	0.02

**Table 3:** Example statistics for randomized controlled trials on vitamin B12 intake and vitamin B12 status

**Reference**		**Observed univariate statistics**					**Required statistics**			
		X_0_*	**Type Y_0_**	**Y_0_**	**n**	**mX**	**mY**	**sY**	**bYX**	**se(bYX)**
[16]	intervention	405	mean, SD	379, 189	17	6.00	5.83	0.47	0.12	0.03
	control	5	mean, SD	211, 77	17	1.61	5.29	0.35		
[17]	intervention	505	med, IQR	198, 158-271	20	6.22	5.29	0.40	0.13	0.04
	control	5	med, IQR	110, 73-165	20	1.61	4.70	0.60		

*) X_0_ represents the dose provided plus the dietary intake. When dietary intake of vitamin B_12_ was not reported 5 μg/day was added to the provided dose. The 5 μg/day was calculated as the average dietary intake from several studies.

## Discussion

The investigated means, standard deviations, correlation coefficients and sample sizes were based on real-life values. The univariate statistics that are investigated in this paper were limited to mean and SD, median and IQR or range and geometric mean and 95% CI. These do not represent all reporting options that can be encountered in the literature, but cover most published papers. Other combinations of univariate statistics that were seen are for example mean with IQR, mean with range, and geometric mean with standard deviation. Also, the investigated regression and correlation coefficients are limited in this paper to those on the absolute or logarithmic scale, whereas sometimes other transformations to normality have been used in reports, such as a square root transformation. However, as the logarithmic transformation is by far the most often used transformation in papers in the medical research area, the equations in this paper will cover most published papers in this field.

The bivariate normal linear model on the logarithmic scale is an approximation that is used here because the data are positive data. Note that it allows the relationship between X_0_ and Y_0_ to be a linear, monotonic convex or monotonic concave function (i.e., for a slope equal, higher or lower than one, respectively). Even though some randomized controlled trials may investigate the dose–response relationship by providing multiple dosages in their study, most of these studies include only one intervention and one control group and consequently it is often unknown what the true relationship is. Therefore, this approximation provides a practical methodology to estimate the dose–response relationship and to combine the results from randomized controlled trials and observational studies. It was outside the scope of the simulation study to investigate other shapes of the dose–response relation.

The transformations in this paper consider reported regression and correlation coefficients that are unadjusted for other variables. It is possible to adjust the equations for adjusted regression or correlation coefficients, if these adjustments were done on the log-scale. However, most often adjustment has been done on another scale, and moreover studies do not report all required statistics. Therefore, we did not consider adjusted coefficients.

In this paper we presented a methodology that allows for information from RCTs and observational studies to be summarised in comparable statistics. One possible application is to combine results of both types of study in a single meta-analysis. In general, a meta-analysis should include as much information as possible. However, there may be systematic differences between observational studies and randomized controlled trials. Therefore, it is advisable to check whether the size of the estimated regression coefficient differs between these different study designs. This may be done by stratified analysis or by using meta-regression techniques.

## Conclusions

The presented methodology provides calculations to use results from published literature to estimate the slope of the dose–response relation incorporating information from both randomized controlled trials and observational studies. The simulations clearly show that there is no observable bias associated with the transformations. Also, it can be seen that when a regression coefficient is reported, it is preferable to report the univariate statistics as mean and SD or geometric mean and 95% CI rather than as median with IQR or range.

## Abbreviations

b: Regression coefficient; CI: Confidence interval; gm: Geometric mean; IQR: Interquartile range; m: Mean; med: Median; r: Correlation coefficient; s: Standard deviation; se: Standard error.

## Competing interests

The authors declare that they have no competing interests’.

## Authors’ contributions

OS participated in the design of the simulation study, performed the statistical analysis and drafted the manuscript. CD helped to draft the manuscript and participated in the design of the simulation study. PvtV participated in the coordination of the study and revised the manuscript critically. HvdV conceived of the study, helped with the statistical analysis and interpretation of the data and revised the manuscript critically. All authors read and approved the final manuscript.

## Authors’ information

OS and CD are both postdoctoral research fellows at the Division of Human Nutrition of Wageningen University, the Netherlands. PvtV is professor of Nutrition and Epidemiology at the Division of Human Nutrition, the Netherlands. HvdV is statistician at Biometris, Wageningen University and Research centre, the Netherlands.

## Pre-publication history

The pre-publication history for this paper can be accessed here:

http://www.biomedcentral.com/1471-2288/12/57/prepub
